# A Comparative Study on the Oxidation of Label-Free Silver Triangular Nanoplates by Peroxides: Main Effects and Sensing Applications

**DOI:** 10.3390/s20174832

**Published:** 2020-08-27

**Authors:** Aleksei Furletov, Vladimir Apyari, Alexey Garshev, Stanislava Dmitrienko

**Affiliations:** 1 Department of Chemistry, Lomonosov Moscow State University, Leninskie Gory, 119991 Moscow, Russia; apyari@mail.ru (V.A.); alexey.garshev@gmail.com (A.G.); dmitrienko@analyt.chem.msu.ru (S.D.); 2 Department of Materials Science, Lomonosov Moscow State University, Leninskie Gory, 119991 Moscow, Russia

**Keywords:** hydrogen peroxide, label-free detection, local surface plasmon resonance, nanoparticles, organic peroxides, silver triangular nanoplates, spectrophotometry

## Abstract

Nowadays, analytical systems based on silver triangular nanoplates (AgTNPs) have been shown as good prospects for chemical sensing. However, they still remain relatively poorly studied as colorimetric probes for sensing various classes of compounds. This study shows that these nanoparticles are capable of being oxidized by peroxides, including both hydrogen peroxide and its organic derivatives. The oxidation was found to result in a decrease in the AgTNPs’ local surface plasmon resonance band intensity at 620 nm. This was proposed for peroxide-sensitive spectrophotometric determination. Five peroxides differing in their structure and number of functional groups were tested. Three of them easily oxidized AgTNPs. The effects of a structure of analytes and main exterior factors on the oxidation are discussed. The detection limits of peroxides in the selected conditions increased in the series peracetic acid < hydrogen peroxide < *tert*-butyl hydroperoxide, coming to 0.08, 1.6 and 24 μmol L^−1^, respectively. *tert*-Butyl peroxybenzoate and di-*tert*-butyl peroxide were found to have no effect on the spectral characteristics of AgTNPs. By the example of hydrogen peroxide, it was found that the determination does not interfere with 100–4000-fold quantities of common inorganic ions. The proposed approach was successfully applied to the analysis of drugs, cosmetics and model mixtures.

## 1. Introduction

It is well known that hydrogen peroxide and the associated active forms of oxygen (anion radicals, hydroxyl radicals, etc.) play an important role in biochemistry, medicine and environmental monitoring [1,2,3,4]. Their excess relative to normal values may indicate the presence of an inflammatory process in the human body, deactivation of mitochondrial biochemical cycles and other important problems [5,6].

To date, many analytical approaches to the qualitative and quantitative determination of hydrogen peroxide have been developed. The attention of most researchers is mainly focused on the enzymatic methods, which have both good sensitivity and good selectivity [7,8]. Along with the undoubted advantages, the methods of this group have a significant drawback associated with the instability of enzymes. Being substances of a protein nature, they are extremely sensitive to environmental parameters, such as temperature, pH and ionic strength, which requires the strictly controlled conditions and greatly complicates the procedure of analysis. In addition, to the best of our knowledge, the above-mentioned approach is limited only to hydrogen peroxide and is not applicable to the determination of organic peroxides. Thus, the development of simple, highly sensitive, cheap and express methods for the determination of peroxides as well as the search for correlations between the structure of analytes and their detectability are important tasks.

Noble metal nanoparticles, in particular silver nanoparticles, are widely proposed in chemical analysis for the determination of both organic and inorganic compounds with various functional groups using spectral and colorimetric methods [9,10,11,12,13,14]. The vast majority of studies considered the problems of the synthesis and application of various analytical systems based on isotropic silver nanoparticles [15,16,17,18,19,20,21,22,23,24]. Fewer articles were connected with discussing the applications of anisotropic silver nanoparticles, in particular, silver triangular nanoplates (AgTNPs) and different composite materials based on them [25,26,27,28,29,30,31,32,33].

Unusual optical properties of silver triangular nanoplates can be explained by the local surface plasmon resonance (LSPR). Relatively large extinction values of silver triangular nanoplates dispersed in an aqueous solution, as well as the possibility of significantly changing their optical properties in the presence of peroxides, allow for considering analytical systems based on AgTNPs as a versatile tool for the detection of hydrogen peroxide [34,35], devoid of many disadvantages of enzymes.

In the present study, the oxidation of label-free AgTNPs by hydrogen peroxide and its organic derivatives was assessed to outline possibilities of this nanoreagent as a colorimetric probe for sensing peroxides.

## 2. Materials and Methods

### 2.1. Reagents and Instruments

In the present study, we used the following reagents: acetic acid (IREA 2000, pure grade), sodium citrate (Sigma-Aldrich, St. Louis, Missouri, USA, ≥99.5%), *tert*-butyl peroxybenzoate (TBPB) (Sigma-Aldrich, 98%), sodium hydroxide (Reachim, pure grade), sodium borohydride (Acros Organics, Belgium, Germany, 99%), *tert*-butyl hydroperoxide (TBHP) (Sigma-Aldrich, 70 wt.% in H_2_O, 99%), poly(N-vinyl-2-pyrrolidone) (Acros Organics, M.W. 58,000 g mol^−1^, 99%), di-*tert*-butyl peroxide (DTBP) (Sigma-Aldrich, 98%), hydrogen peroxide (HP) (Sigma-Aldrich, 30 wt.% in H_2_O, ACS), peracetic acid (PAA) (Sigma-Aldrich, 39 wt.% in glacial acetic acid, pure grade) and silver nitrate (PZTsM-Vtormet, analytical grade). In all experiments, the Millipore Simplicity water purification system was used to obtain deionized water (Merck Millipore, Burlington, MA, USA).

Absorption spectra of AgTNPs in a wavelength range of 400–1100 nm were recorded using an SF-104 spectrophotometer (Aquilon, Nakhodka, Russia). Stirring of solutions was carried out using an Ekros PE-6100 magnetic mixer (Ekros, Novocherkassk, Russia). pH values were measured using an Ekspert 001 pH-meter (Ekoniks Ekspert, Moscow, Russia).

Transmission electron microscope (TEM) images of silver triangular nanoplates were recorded with the Libra 200 microscope (Carl Zeiss, Oberkochen, Germany) using the following technique. Samples of the studied nanoparticles were deposited onto a copper grid support which had been previously coated with a formvar film covered with amorphous carbon. These samples were further dried in air for 20–30 min and then in vacuum using the Turbo Pumping Station Model 655 system (Gatan, Pleasanton, CA, USA) for 10–12 h. All measurements of electrokinetic potentials of the colloidal systems based on silver triangular nanoplates were performed with the Zetasizer Nano ZS light scattering system (Malvern Instruments, Malvern, UK).

### 2.2. Synthesis of Silver Triangular Nanoplates in Aqueous Solution

**Figure 1:**
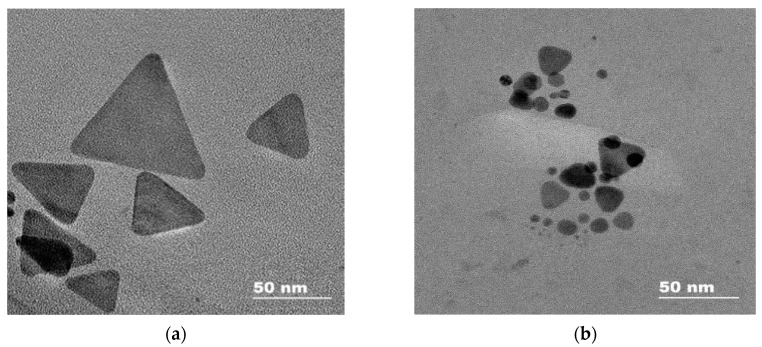
A TEM image of silver triangular nanoplates (AgTNPs) before (**a**) and after (**b**) the interaction with hydrogen peroxide. *c* (AgTNPs) = 0.16 mM Ag, *c* (HP) = 30 μM, pH 6.

Synthesis of label-free silver triangular nanoplates was performed according to the method described earlier in the literature [36]. First of all, 2.30 mL of sodium citrate solution (1%), 0.60 mL of poly(N-vinyl-2-pyrrolidone) solution (20 mg mL^−1^) and 1.20 mL of hydrogen peroxide solution (3%) were successively added to 4.80 mL of AgNO_3_ solution (1 mM) under vigorous stirring. Then, 1.00 mL of NaBH_4_ solution (0.035 M) was added into that mixture. After half an hour, when the solution changed its color from yellow to blue, the stirring was stopped. The concentration of silver triangular nanoplates in the resulting colloidal solution was found to be equal to 0.52 mM in terms of atomic silver. A TEM image of AgTNPs is shown in Figure 1a.

### 2.3. Procedures

To plot the kinetic curves of the interaction between AgTNPs and the studied peroxides, certain amounts of peroxides were added into test tubes. After that, 1.50 mL of AgTNPs solution (0.52 mM) was added into each test tube as well. The final volume of each reaction mixture was adjusted up to 5.00 mL using the acetate buffer solution (pH 6.0) as it was found not to adversely affect optical properties of AgTNPs and not to interfere with the determination of peroxides. Absorption spectra of AgTNPs were recorded 1, 5, 10, 15, 20 and 25 min after the last reagent was added.

The effect of pH on the analytical response was evaluated by the following procedure. First of all, a certain amount of the determined compound was introduced into the test tubes. After that, 1.5 mL of 0.52 mM AgTNPs solution was added. The final volume was adjusted up to 5.0 mL with 0.1 M acetic acid and 0.1 M sodium hydroxide solutions mixed with each other in various ratios. After a certain time required to achieve the maximum analytical response, absorption spectra were recorded.

To plot the calibration curves, 1.50 mL of AgTNPs solution (0.52 mM) and different amounts of peroxides were added into test tubes. The final volume of each reaction mixture was adjusted up to 5.00 mL using the acetate buffer solution (pH 6.0). Absorption spectra of AgTNPs were recorded after 5 min in the case of HP, 15 min in the case of PAA, and 20 min in the case of TBHP.

## 3. Results and Discussion

### 3.1. Interaction of Peroxides with Label-Free Silver Triangular Nanoplates

Interaction of peroxides with AgTNPs as well as with other nanophases is a quite complex process. It may include adsorption onto the nanoparticles, electrostatic interactions, and redox reactions with both peroxides and the reactive oxygen species produced from them. In addition, the above-mentioned interaction is also able to change the state of AgTNPs in solution and, therefore, can affect both LSPR and SERS effects on the surface of nanoparticles [37]. In this sense, oxidation activity and structure of a peroxide may drastically affect its ability to interact with AgTNPs. As a result, analytical features of merit may significantly vary for different peroxides. In this study, five peroxides differing in their oxidation activity and structure of the substituents were studied. Apart from hydrogen peroxide, two hydroperoxides (TBHP, PAA) and two fully substituted peroxides (DTBP, TBPB) were chosen (Table 1).

**Figure 2:**
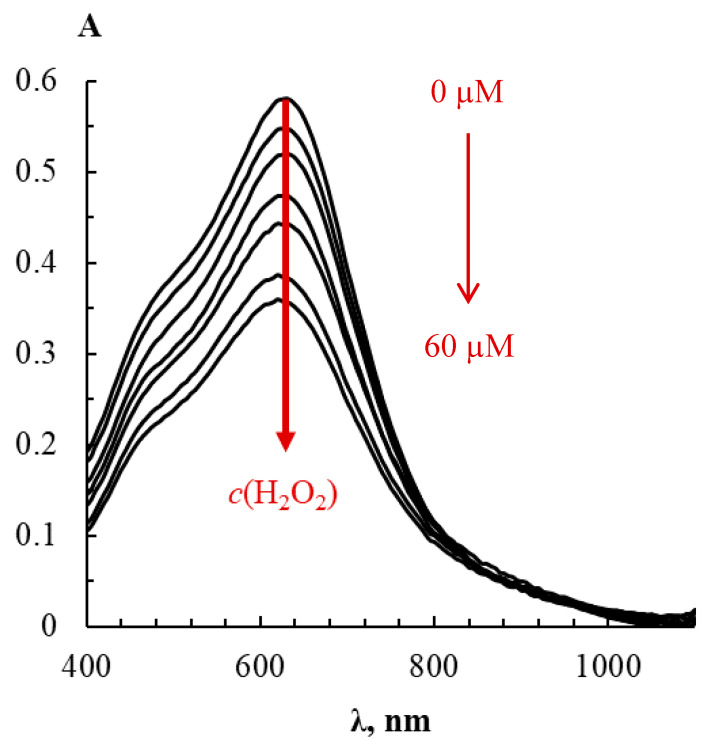
Absorption spectra of AgTNPs colloidal solutions at various concentrations of hydrogen peroxide. *c* (AgTNPs) = 0.08 mM Ag, *c* (HP) = 0–60 μM, pH 6, *t* = 10 min.

Some important features of AgTNPs oxidation by these peroxides were evaluated. It was found that the interaction of AgTNPs with HP and PAA leads to a remarkable decrease in the LSPR band intensity at 620 nm (Figure 2), which is especially noticeable in the case of PAA. In all experiments, we considered the value of change in the absorbance of silver triangular nanoplates solutions at their absorption band maximum (ΔA) as the analytical signal.

**Figure 3:**
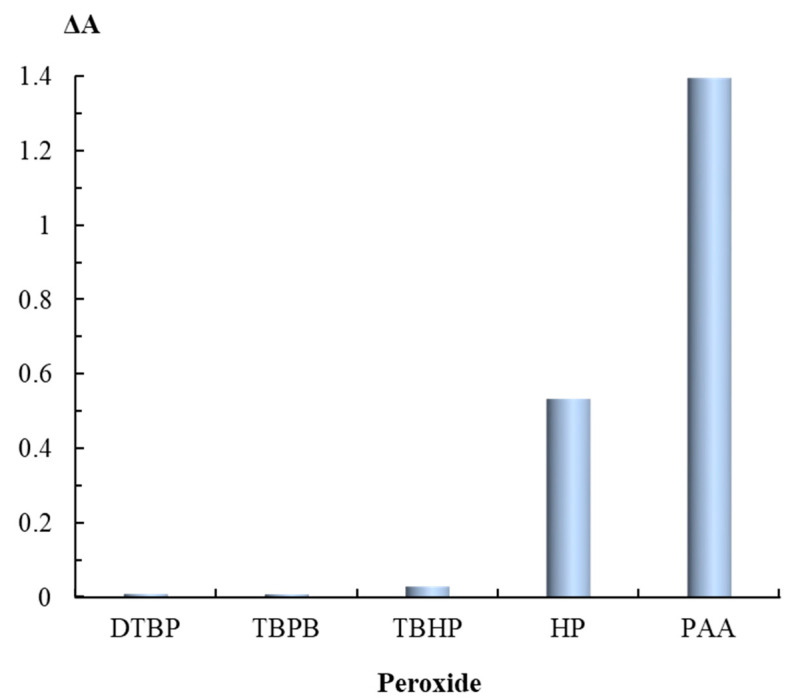
Change in the absorbance of AgTNPs solutions depending on the nature of a peroxide. *c*(AgTNPs) = 0.16 mM Ag, *c*(peroxide) = 0.1 mM, pH 6, *t* = 10 min.

TEM images of nanoparticles after their interaction with HP (Figure 1b) show a very small number of silver nanospheres and nanodisks as well as triangular nanoplates with rounded edges. All particles have a size lower than the initial AgTNPs, which is probably a result of their partial oxidation and dissolution. In the case of TBHP containing a bulky electron-donor *tert*-butyl radical, the changes are manifested to a much lesser extent. The di-substituted peroxides, TBPB and DTBP, do not affect AgTNPs, regardless of the electron-acceptor properties of the substituents (Figure 3). These facts indicate that the oxidation of AgTNPs is promoted by a decrease in the size of substituents in a peroxide molecule and an increase in their electron-acceptor properties determined by the values of inductive and mesomeric effects of functional groups of the above-mentioned peroxides. It should be also highlighted that the observed consistent pattern is in good agreement with the values of the oxidation potential of the above peroxides. Indeed, a decrease in the size of substituents in a peroxide molecule, as well as an increase in their electron-acceptor properties, leads to an increase in the oxidation potential of peroxides, which contributes to the easier oxidation of nanoparticles.

The observed spectral changes can be considered as the basis of novel spectrophotometric methods for the determination of peroxides. Therefore, the effects of exterior factors such as pH, time, and concentration of AgTNPs should be considered to achieve the best analytical performance of the peroxides determination.

#### 3.1.1. Effect of pH

**Figure 4:**
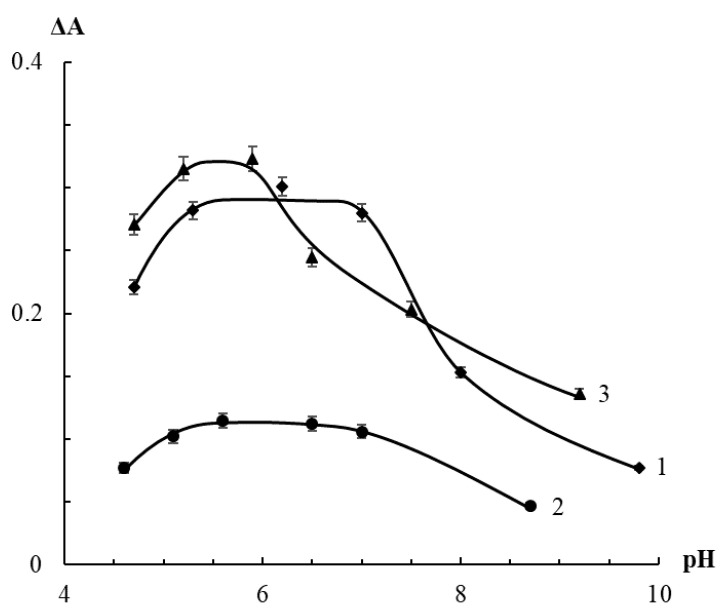
Change in the absorbance of AgTNPs solutions in the presence of hydrogen peroxide (1), *tert*-butyl hydroperoxide (2) and peracetic acid (3) depending on pH. *c* (AgTNPs) = 0.16 mM Ag; (1) *c* (HP) = 50 μM, *t* = 10 min; (2) *c* (TBHP) = 300 μM, *t* = 20 min; (3) *c* (PAA) = 3 μM, *t* = 15 min.

The dependence of AgTNPs absorbance on pH in the presence of HP, TBHP and PAA was evaluated. The results are represented in Figure 4. It was found that the maximum analytical response is observed in the pH range of 5–7 in the case of both HP and TBHP, and 5–6 in the case of PAA. A decrease in the change of AgTNPs absorbance with increasing pH value is probably associated with a decrease in the oxidation potential of peroxides in alkaline medium, which is most clearly observed for PAA due to its electrolytic dissociation, as well as with a decrease in the activity of the nanoparticles themselves. A decrease in the signal at pH < 5 is observed regardless of the nature of a peroxide, since it is associated with the instability of AgTNPs under these conditions [38].

#### 3.1.2. Effect of the Interaction Time

**Figure 5:**
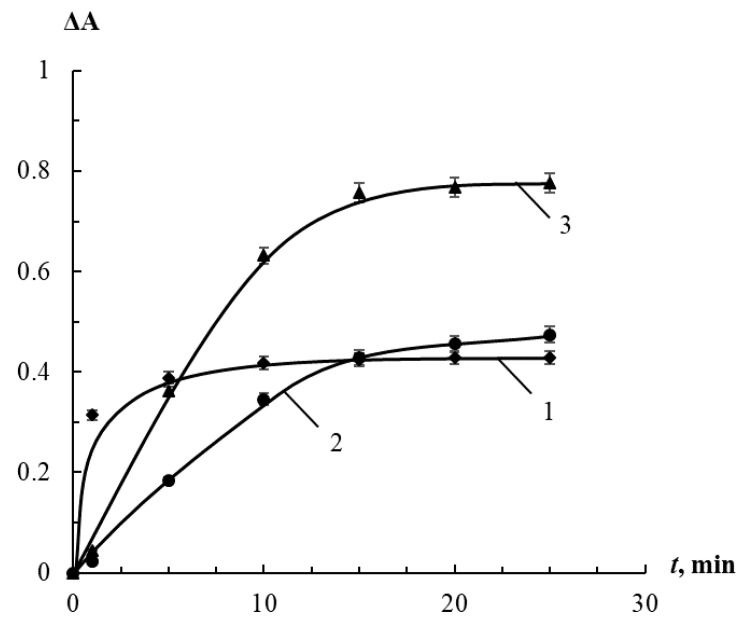
Change in the absorbance of AgTNPs solutions in the presence of hydrogen peroxide (1), *tert*-butyl hydroperoxide (2) and peracetic acid (3) depending on the interaction time. *c* (AgTNPs) = 0.16 mM Ag, pH 6. (1) *c* (HP) = 70 μM; (2) *c* (TBHP) = 1200 μM; (3) *c* (PAA) = 7.5 μM.

The influence of the interaction time on sensitivity of the peroxides determination was studied. It was found that the maximum intensity change of the solutions can be observed in 5 min after mixing the reagents in the case of HP, 15 min in the case of PAA, and 20 min in the case of TBHP (Figure 5). It is likely that an increase in the size of the radical R in the molecules of hydroperoxides ROOH contributes to a decrease in the rate of their interaction with AgTNPs because of the steric effects.

#### 3.1.3. Effect of AgTNPs Concentration

**Figure 6:**
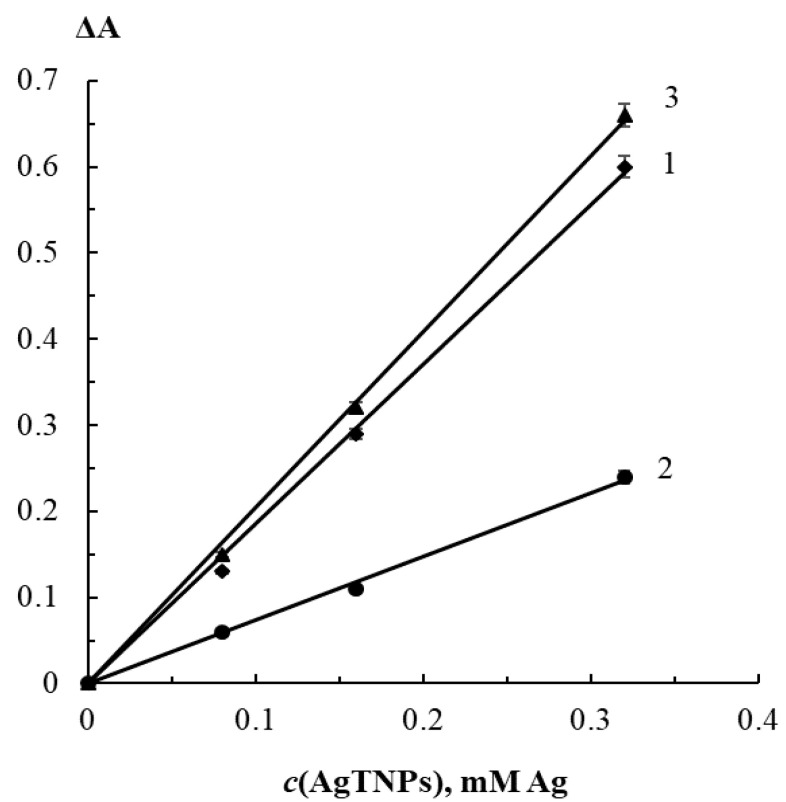
Change in the absorbance of AgTNPs solutions in the presence of 50 μM hydrogen peroxide (1), 300 μM *tert*-butyl hydroperoxide (2) and 3 μM peracetic acid (3) depending on the AgTNPs concentration. pH 6, t = 20 min.

The effect of AgTNPs concentration on the analytical response was evaluated as well. It was found that with an increase in AgTNPs concentration from 0 up to 0.32 mM Ag, sensitivity of the determination of peroxides increases (Figure 6). It should be stressed, however, that measurement errors of the analytical response increase sympathetically. For further experiments, a AgTNPs concentration of 0.16 mM Ag was chosen, which corresponds to the middle of the above-mentioned concentration range.

### 3.2. Spectrophotometric Determination of Peroxides

#### 3.2.1. Analytical Performance

All found relations and effects should be taken into account when assessing possibilities of AgTNPs application for spectrophotometric determination of hydrogen peroxide and its organic derivatives. In this study, we have estimated the main analytical features of such an approach. Limits of detection (LODs) and limits of quantitation (LOQs) were evaluated using 3σ and 9σ criteria, respectively. Some analytical characteristics of the approach proposed in the present article are shown in Table 2. It was found that the limits of peroxide detection in the selected optimal conditions increase in the series peracetic acid < hydrogen peroxide < *tert*-butyl hydroperoxide and come to 0.08, 1.6, and 24 μmol L^−1^, respectively. In all cases, the determination ranges were about the order of magnitude. The relative standard deviations (RSDs) calculated for concentrations corresponding to midpoints of the determination ranges do not exceed 3%. This indicates good reproducibility of the analysis.

#### 3.2.2. Selectivity Studies

Selectivity of the proposed method was evaluated by the example of HP determination in the presence of a number of cations and anions. It was found that the determination of 30 μmol L^−1^ HP is not affected at least by 4000-fold quantities of Na^+^, K^+^, CH_3_COO^−^, 1000-fold quantities of Mg^2+^, Ca^2+^, Ba^2+^, Al^3+^, NO_3_^−^ and 100-fold quantities of Pb^2+^, Cu^2+^ and Cl^−^. However, the determination is affected by 50-fold quantities of Fe^3+^, Ni^2+^, Cr^3+^ and 10-fold quantities of Br^−^ and I^−^.

#### 3.2.3. Sample Analysis

In order to show the applicability of the proposed method to the analysis of samples, the determination of various peroxides using AgTNPs was performed after appropriate dilution of the samples. The following samples were used: “Hydroperit” formulation (Tatkhimpharmpreparaty, Kazan, Russia) and “Estel De Luxe” hair oxygent (Unicosmetik, Saint-Petersburg, Russia) containing HP as an active component. Model systems based on *tert*-butanol spiked with TBHP were also analyzed, which simulate contamination of the chemical reagent with the peroxide product of its oxidation. To confirm the accuracy of HP determination, the “Hydroperit“ formulation and “Estel De Luxe” hair oxygent were alternatively analyzed by permanganometric titration with visual detection of the equivalence point. The accuracy of TBHP determination in *tert*-butanol was assessed using the standard addition method. The results are represented in Table 3 and Table 4. One can see that the results of HP determination coincide with the data provided by manufacturers and the results of the alternative method. In the case of TBHP determination, the found peroxide content matches the added amount. This indicates good accuracy of the determination. RSD values do not exceed 8%, which proves good reproducibility of the AgTNPs-based method.

#### 3.2.4. Comparison with Other Methods for the Determination of Peroxides

Some analytical features of other reported methods for peroxides determination are represented in Table 5. One can conclude that the approach proposed in the present study has rather good sensitivity as long as our method makes it possible to detect both peracetic acid and hydrogen peroxide, with several times lower limits of detection than many of the reported methods. Unfortunately, we have failed to find any papers on the determination of TBHP, so it seems to be impossible to compare the calculated LOD with published data in this case. The most sensitive reported methods of PAA and HP determination have LODs of about two or three times better than was achieved with AgTNPs. Nonetheless, they have a number of disadvantages mainly associated with the long duration and high cost of a single analysis. Contrary, the technique proposed in the present study utilizes rather simple equipment and requires a short time for a single analysis as well as seeming to be reasonably selective and sensitive.

## 4. Conclusions

As shown in the present article, AgTNPs are a promising colorimetric probe for sensing hydrogen peroxide and its organic derivatives. It has been found that AgTNPs are capable of being oxidized by both hydrogen peroxide and organic hydroperoxides decreasing the LSPR band intensity at 620 nm. Di-substituted peroxides do not affect AgTNPs. It has been suggested that the oxidation is promoted by a decrease in the size of a substituent in hydroperoxide and an increase in its electron-acceptor properties. The interaction proceeds at pH from 5 to 6–7 in 5–20 min depending on the nature of the peroxide. The detection limits of peroxides in the selected conditions increase in the series peracetic acid < hydrogen peroxide < *tert*-butyl hydroperoxide and come to 0.08, 1.6 and 24 μmol L^−1^, respectively. It was found that the determination of hydrogen peroxide does not interfere with 100–4000-fold quantities of common inorganic ions. The advantages of the AgTNPs-based method include simplicity, rapidity, good analytical performance, availability of the equipment and ease of the test-method’s implementation.

## Figures and Tables

**Table 1:** Peroxides studied in this work.

Chemical Name	MW, g mol^−1^	Structure	Type ^a^
Hydrogen peroxide (HP)	34.01	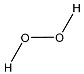	Unsubstituted
*tert*-Butyl hydroperoxide (TBHP)	90.12	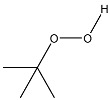	Monosubstituted, EDS
Peracetic acid (PAA)	76.05	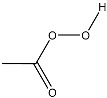	Monosubstituted, EAS
di-*tert*-Butyl peroxide (DTBP)	146.23	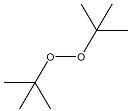	Disubstituted, EDS/EDS
*tert*-Butyl peroxybenzoate (TBPB)	194.23	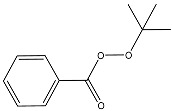	Disubstituted, EDS/EAS

^a^ EDS—electron-donor substituent, EAS—electron-acceptor substituent.

**Table 2:** Analytical features of the peroxides determination.

Analyte	*k*, L mmol^−1^	*r* ^2^	LOD, μmol L^−1^	Determination Range, μmol L^−1^	RSD ^a^, %	RSD ^b^, %
PAA	106	0.990	0.08	0.25–6	3	11.3
HP	5.70	0.991	1.6	5–60	2	10.5
TBHP	0.376	0.990	24	72–600	3	11.1

^a^ Calculated for the middle of the determination range; ^b^ Calculated for the LOQ value.

**Table 3:** Determination of hydrogen peroxide in samples (*n* = 3, *p* = 0.95).

Sample	Content of Hydrogen Peroxide, wt.%					*t*-test Value ^c^
	Declared by Manufacturer	AgTNPs-Based Method		Alternative Method		
		Found	RSD%	Found	RSD%	
Hydroperite formulation ^a^	36.2	36 ± 4	5	36 ± 2	2	0
Estel De Luxe hair oxygen ^b^	9.0	8.8 ± 0.9	4	9.3 ± 0.6	2	1.98

^a^ Composition of the formulation (per one tablet): 542.55 mg of HP, 957.45 mg of urea; ^b^ Composition of the sample: HP, cetearyl alcohol, ceteareth-20, cetrimonium chloride, EDTA, phosphoric acid, sodium stannate; ^c^ Threshold t-test value (*p* = 0.95, *f* = 4) is 2.78.

**Table 4:** Determination of *tert*-butyl hydroperoxide in a model sample (*n* = 3, *p* = 0.95).

Sample	Content of *tert*-Butyl Hydroperoxide, mg g^−1^		RSD%
	Added	Found	
Model mixture based on *tert*-butanol spiked with TBHP	0	0	—
	1.2	1.1 ± 0.2	8

**Table 5:** Comparison of AgTNPs-based method with other methods for peroxides determination.

Analyte	Method	LOD, μmol L^−1^	Determination Range, μmol L^−1^	Reference
PAA	Spectrophotometry	0.6	0.6–100	[39]
	Fluorimetry	0.04	0.1–20	[40]
	Present method	0.08	0.25–6	This article
HP	Spectrophotometry	1000	1000–100,000	[21]
	Visual colorimetry	1000	1000–10,000	[41]
	Spectrophotometry	80	500–15,000	[3]
	Spectrophotometry	18	29–150; 290–590	[22]
	Light diffraction	8	10–670	[42]
	Voltammetry	2	5–600	[43]
	Sweep voltammetry	0.5	1–300	[44]
	Spectrophotometry	0.00136	0.05–1	[34]
	Spectrophotometry	0.00037	10–500	[35]
	Present method	1.6	5–60	This article
